# The Effect of Filler Dimensionality and Content on Resistive Viscoelasticity of Conductive Polymer Composites for Soft Strain Sensors

**DOI:** 10.3390/polym15163379

**Published:** 2023-08-11

**Authors:** Quanyi Mu, Ting Hu, Xinya Tian, Tongchuan Li, Xiao Kuang

**Affiliations:** 1 School of Physics, Ningxia University, Yinchuan 750021, China; 12022130631@stu.nxu.edu.cn (T.H.); tianxinya2@163.com (X.T.); litongchuan@boe.com.cn (T.L.); 2 Ningxia Key Laboratory of Intelligent Sensing for Desert Information, Ningxia University, Yinchuan 750021, China; 3 Division of Engineering in Medicine, Brigham and Women’s Hospital, Harvard Medical School, Cambridge, MA 02139, USA

**Keywords:** resistance relaxation, resistive hysteresis, dynamic stability, electro-mechanical properties, conductive network

## Abstract

Soft strain sensors based on conductive polymer composites (CPCs) provide a simple and feasible detection tool in wearable electronics, soft machines, electronic skin, etc. However, the CPCs-based soft strain sensors exhibit resistive viscoelasticity (or time-dependent properties) that hinder the intuitive reflection of the accurate strain and a simple calibration process. In this paper, CPCs with different carbon nanotubes (CNTs) and carbon black (CB) contents were prepared, and electro-mechanical experiments were conducted to study the effect of filler dimensionality and content on the resistive viscoelasticity of CPCs, aimed at guiding the fabrication of CPCs with low resistive viscoelasticity. Furthermore, resistive viscoelasticity and mechanical viscoelasticity were compared to study the origin of the resistive viscoelasticity of CPCs. We found that, at the vicinity of their percolation threshold, the CPCs exhibit high resistive viscoelasticity despite their high sensitivity. In addition, the secondary peaks for CB/SR composite were negligible when the CB concentration was low. Generally, compared with one-dimensional CNT-filled CPCs, the zero-dimensional CB-filled CPCs show higher sensitivity, lower resistive hysteresis, lower resistance relaxation ratio, and better cyclic performance, so they are more suitable for sensor usage. By comparing the resistive viscoelasticity and mechanical viscoelasticity of CPCs, it is indicated that, when the concentration of nanoparticles (NPs) approaches the percolation thresholds, the resistive viscoelasticity is mainly derived from the change of conductive network, while when the concentration of NPs is higher, it is primarily due to the unrecoverable deformations inside the material.

## 1. Introduction

Soft strain sensors are widely used in the biomedical field, soft machines, and wearable electronics [1]. Conductive polymer composites (CPCs), often combining excellent mechanical and electrical properties with low fabrication costs, are important in soft strain sensing [2]. CPCs are usually fabricated by filling different dimensional conductive particles, such as zero-dimensional carbon black (CB) and silver nanoparticles, one-dimensional carbon nanotubes (CNTs), and two-dimensional graphene into the insulating polymer matrix, such as silicone elastomers, thermoplastic elastomers, and natural rubber [3,4]. Among them, silicone rubber has good durability, excellent solvent/chemical resistance, wide service temperature and high thermal stability, superior biocompatibility, low curing temperature, and non-toxicity, which make it a good candidate for wearable electronic and soft sensors [1,5,6]. In CPCs-based strain sensors, the insulating polymer matrix provides flexibility and stretchability, while the conductive materials construct the conductive sensor network. Due to the viscoelastic nature of the polymer matrix, the CPCs-based strain sensors exhibit resistive viscoelasticity properties [7,8,9], such as rate dependence [10,11], relaxation [12,13], hysteresis [14,15], and dynamic resistance response [16]. Resistive viscoelasticity affects performance parameters such as linearity, sensitivity, repeatability, cycle stability, etc., which are critical for evaluating strain sensor materials [17,18,19]. Thus, resistive viscoelasticity remains a challenge that hinders the accurate measurements of soft resistive sensors [20].

To accurately predict and calibrate the soft resistive sensors, modeling is an effective way to reveal the underlying mechanism and predict resistance drift or decay of CPCs. Based on the dynamic cyclic loading experimental results, Mersch et al. developed a model that incorporates the visco-elastic nature of the polymer and representations of the percolative networks in the strain direction and the transversal direction, aiming to optimize sensor systems [16]. Similarly, a micromechanical piezoresistive model study showed that the time-dependent transverse deformation leads to network compaction and is the mechanism behind resistance relaxation and hysteresis [21]. Combing the tunneling theory, a multi-branch model was developed, which used only a single set of parameters to predict the resistance relaxation behaviors of CPCs under different strains and different loading rates [11]. Zhang et al. found that resistance drift/decay for most CPCs under quasi-static and dynamic loadings is a consequence of the relaxation of both the polymer chains and the conductive network [22].

In addition to model prediction, reducing mechanical viscoelasticity experimentally turned out to be an effective method for minimizing the resistive viscoelasticity of CPCs. Among them, the straightforward approach is to use a substrate/matrix with smaller mechanical hysteresis, which generally results in lower electro-mechanical hysteresis and better repeatability of sensors [14]. In addition, designing a special structure or coating is also an effective way to minimize the resistive viscoelasticity of CPCs. For instance, Cui et al. designed a stretchable rough filament strain sensor with a dentate groove structure to eliminate the shoulder peak and improve recoverability [23]. Inspired by the growth ring of a tree, Kong et al. prepared a highly stretchable silver nanowire/polydimethylsiloxane-based fibrous strain sensor with a spiral structure with negligible hysteresis and excellent reliability [24]. In another work, a bioinspired liquid-filled cell-type structural pressure sensor was designed with a minimal resistance hysteresis of 7.7% [20]. Qu et al. coated CB/nitrile rubber composite with polydopamine to preserve the CB layer, and fabricated a flexible strain sensor, which has prominent robustness under cyclic strain sensing tests [2].

The type and content of fillers influence the viscoelastic behavior of the elastomer materials. For example, an increased filler fraction in rubbers leads to higher mechanical hysteresis [25]. Correspondingly, filler content [26,27], properties [28], dimensionality [29], and aspect ratio [30] influence the electrical filler network structure, which in turn affects the electro-mechanical performance of CPCs. Many efforts have been devoted to improving the electrical filler network structure to obtain a good electrical performance of CPCs, for instance, by using ionic liquid to help CPCs suppress the effect of filler arrangement to improve the resistive response independent of strain rate [31]. The presence of the miscible polymer poly(methyl methacrylate) (PMMA) results in a modified distribution of the CNT networks and makes it stable in cyclic testing [26]. Compared with the solution and flocculation methods, the two-roll method produced the best MWCNTs distribution, and the ‘shoulder peak’ phenomenon has not been observed in the composite prepared by this method [32]. Due to the synergistic effect of CNTs and CB, CPCs can obtain good recoverability under repeated stretching/releasing loads [33,34].

Although varying the filler content and filler type is a more convenient way to adjust the electrical filler network’s structure to optimize the electric performance of CPCs, to date, systematic research on the effect of filler content and dimensionality on the resistive viscoelasticity of CPCs is lacking. In this paper, we systematically investigated the effect of zero-dimensional CB and one-dimensional CNTs content on the resistive viscoelasticity of CPCs. The paper is arranged as follows. We first introduce the materials and experimental methods. By studying the electrical properties and scanning electron microscopy (SEM) images, we confirmed the good dispersion of CNT and CB fillers in the silicone rubber matrix. Then, we investigated the resistive viscoelasticity of CPCs through loaded/unloaded tests, relaxation tests, and dynamic cyclic loading tests. In addition, we analyzed the origin of the resistive viscoelasticity of CPCs.

## 2. Materials and Methods

### 2.1. Materials

Commercial CNTs (HQNANO-010-0, Tanfeng Tech. Inc., Suzhou, China) and CB (BP2000, CABOT, Boston, MA, USA) were used as filler materials for the CPCs for NPs dimensionality comparison. According to the supplier, CNTs were fabricated from a chemical vapor deposition process with a purity > 95 wt%. The dimensions of the CNTs were 8–15 nm in diameter and up to 12 μm in length. CB with a particle size of 15 nm and a density of 144 g/L. Cyclohexane (CYH, Sinopharm Chemical Reagent, Shanghai, China) and polyvinylpyrrolidone (PVP, Tanfeng Tech. Inc., Suzhou, China) were used as received to disperse the CNTs and CB. A commercially available silicone rubber (SR, Tianying, Shenzhen, China) was used as the matrix material for the CPCs.

### 2.2. Experimental Methods

#### 2.2.1. Preparation of CPCs and Strip Specimens

To prepare the carbon nanotubes/silicone rubber (CNT/SR) and carbon black/silicone rubber (CB/SR) CPCs, ultrasonication, a typical mechanical dispersion technique, was used to disperse the CNTs and CB [35]. The weight ratio of the NPs, PVP, and CYH was 1:0.1:100. The desired amount of NPs was dispersed in the CYH/PVP solution by sonication (Scientz-IID, Ningbo Xinzhi Biotechnology Ltd., Ningbo, China) for 10 min at 20 kHz (1000 W, oscillation amplitude 50%). The SR was also ultrasonically dispersed in the same weight of CYH using the same operating conditions as above, and then added to the NPs/PVP/CYH mixture. The above new mixture was stirred at 2000 rpm for 10 min (NE-L, 9nbo, Suzhou, China), then the curing agent for SR was added and ultrasonically dispersed for another 10 min. Finally, the mixture was poured into the polytetrafluoroethylene (PTFE) mold and placed under ventilation conditions at room temperature for at least 6 h to remove the CYH solvent for curing. The fabricated strip specimens with a size of 100 × 10 × 0.8 mm^3^ were used for the electro-mechanical testing. It should be noted that we named the sample with NPs concentration and nanofiller type, e.g., 0.5CNT/SR and 4CB/SR were the CPCs containing 0.5 wt% CNT and 4 wt% CB, respectively.

#### 2.2.2. Electro-Mechanical Behavior Characterization

Resistivity. A sourcemeter (Keithley 2450, Tektronix Inc., Shanghai, China), controlled by its own data acquisition program (Kickstart), was used to measure and record the resistance of the molded samples. A two-point probe method was used to calculate the bulk resistivity and the resistance change during mechanical deformation tests. The resistivity of the molded strips was calculated as follows:

(1) $$\rho =\frac{R\cdot A}{l},$$

where *R* is the measured resistance, *l* is the distance between terminals, and *A* is the cross-section area of the molded specimen.

**Figure 1:**
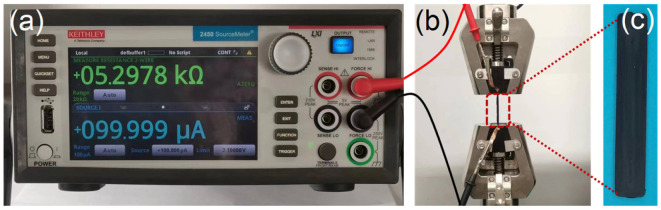
The experimental setup for measuring the electro-mechanical properties of CPCs. (**a**) A sourcemeter was used to measure the resistance change during the mechanical tests. (**b**) An electromechanical testing frame was used to conduct mechanical tests. (**c**) The strip specimens.

As shown in Figure 1, we used the MTS electro-mechanical testing frame (CMT-6104, MTS Industrial Systems (China) Co., Ltd., Shenzhen, China) and the Keithley 2450 sourcemeter to measure the electro-mechanical properties, which was similar to our previous work [36]. For all electro-mechanical tests, the distance between the two clamps was set to about 44 mm. All electro-mechanical property tests were performed at room temperature (~22 °C).

Stress–strain test. For the stress–strain test, the strain control mode was used with a strain rate of 20 mm min^−1^ (0.0076 s^−1^), and a strain rate of 264 mm min^−1^ (0.1 s^−1^) was used for comparison.

Cyclic loading/unloading test. For the loaded/unloaded test, the sample was stretched at a speed of 20 mm min^−1^ (0.0076 s^−1^) to 20% strain, and then released to the initial position at the same speed without holding between stretching and releasing.

Relaxation experiment. In the resistance relaxation experiment, the CPCs were stretched to three different levels of maximum strain (10%, 20%, and 30%) by using a constant extension rate of 132 mm min^−1^ (0.05 s^−1^). The relaxation period for all the relaxation tests was about 1800 s.

Dynamic cyclic loading test. For the dynamic cyclic loading test, the sample was stretched to 20% strain at a speed of 264 mm min^−1^ (0.1 s^−1^), held for 8 s, and then released to the initial strain at the same speed (0.1 s^−1^). The holding time between cycles was 8 s. The resistance change during cyclic testing was also recorded.

#### 2.2.3. Other Characterization

To investigate the microstructure of the CPCs, scanning electron microscopy (SEM, Sigma 300, ZEISS, Oberkochen, Germany) images were taken with an accelerating voltage of 5 kV.

## 3. Results and Discussion

### 3.1. Ensure the Dispersion Quality of NPs by Electrical Properties of CPCs

**Figure 2:**
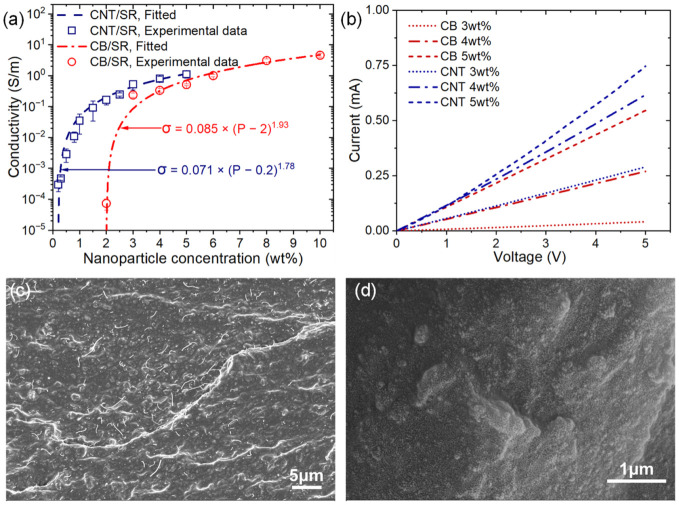
(**a**) Conductivity as a function of NPs loading, and fitted by Equation (2). (**b**) IV curves of CPCs resistors with similar dimensions. SEM images of the fracture surface of (**c**) 4CNT/SR and (**d**) 4CB/SR sample.

Since the preparation process has an effect on the dispersion of NPs and the microstructure morphology of CPCs, this in turn affects the electro-mechanical properties of CPCs [32,37]. We first studied the electrical properties of the ultrasonic blended CPCs before investigating their electro-mechanical properties. The electrical conductivity of the CPCs was measured using the standard two-point probe method, and the function curves of the electrical conductivities and filler contents of different CPCs are compared in Figure 2a. The CNT/SR and CB/SR obtained electrical conductivity at around 0.2 wt% CNT loading and 2 wt% CB loading, respectively, indicating the formation of a conductive percolation network. According to the classical percolation theory, the scaling law between the conductivity of the CPCs and its filler concentration is defined as [7]:

(2) $$\sigma =\sigma_{0}{\left(P-P_{c}\right)}^{t},$$

where σ is the conductivity of the CPCs, $\sigma_{0}$ is the scaling factor, *P* is the filler loading, and *P_c_* is the percolation threshold. The exponent *t* is a parameter related to the dimensionality of the conductive network. The values of *t* are estimated as 1.78 for CNT/SR and 1.93 for CB/SR, respectively, which agree with the calculated value for a 3D random network (*t*~2.0) [17,29].

The percolation thresholds of our CPCs are reasonable as compared to values obtained in other works, such as 0.1 wt%, 0.39 wt%, or 0.4 wt% for CNT-filled composites [17,30,35], and 5.22 wt% for CB-filled composites [33]. It is obvious that the percolation threshold of CB/SR is much higher than that of CNT/SR. Because of their low aspect ratio compared to one-dimensional CNT, a higher content of CB particles is needed to construct interlinked conductive networks [29]. Consequently, the CNT/SR has higher conductivity than that of CB/SR with the same NPs loading.

Figure 2b compares the I–V curves of the CNT/SR and CB/SR samples with the same dimension and various NPs loading. It can be seen that the resistance of the CPCs is in the kilo-ohm to mega-ohm level, so we ignored the contact resistance. The CPCs exhibit obvious ohmic behavior, the resistance decreases with the increasing NPs loading, and the linearity of the curves indicates a stable conductivity. It also indicates that, under the same NPs loading, the conductivity of the CNT/SR composites is higher than that of CB/SR composites. We further examined the dispersion quality of the NPs in CPCs using SEM, and the cross-sectional images of the fractured sample surface are shown in Figure 2c,d. It can be seen that NPs are homogeneously dispersed in the SR matrix, and negligible clusters are observed in the CPCs. The low percolation threshold and stable conductivity characteristics indicate that the prepared CPCs have good NPs dispersion, which is reliable for the subsequent investigation of their electro-mechanical properties.

### 3.2. Effect of Filler Content on Sensitivity and Modulus of CNT/SR and CB/SR

Although in our experiments, the percolation thresholds of CNT/SR and CB/SR composites were 0.2 wt% and 2 wt%, respectively, it was found that, when the CNT loading ≥ 0.5 wt% (CB loading ≥ 3 wt%), the electro-mechanical properties between specimens with the same recipe were closer. Additionally, CPCs became insensitive when NPs loadings were high. Therefore, we conducted electro-mechanical tests on CPCs with CNT loading from 0.5 wt% to 5 wt% and CB loading from 3 wt% to 10 wt%, focusing on the percolation region. The true stress–strain curves and monotonic strain sensing behaviors of CNT/SR and CB/SR are summarized in Figure S1. It should be noted that we selected 0.5CNT/SR, 4CNT/SR, 4CB/SR, and 10CB/SR as the four representative CPCs based on the following three reasons: (i) 0.5CNT/SR and 10CB/SR composites had the lowest and highest NPs loading in the experiment, respectively; (ii) the NPs loading of 4CNT/SR and 4CB/SR samples was the same, but the 4CB/SR was around the percolation region and the 4CNT/SR was far away from the percolation threshold; (iii) the four representative samples belonged to two different filler dimensionalities and different NPs loadings.

**Figure 3:**
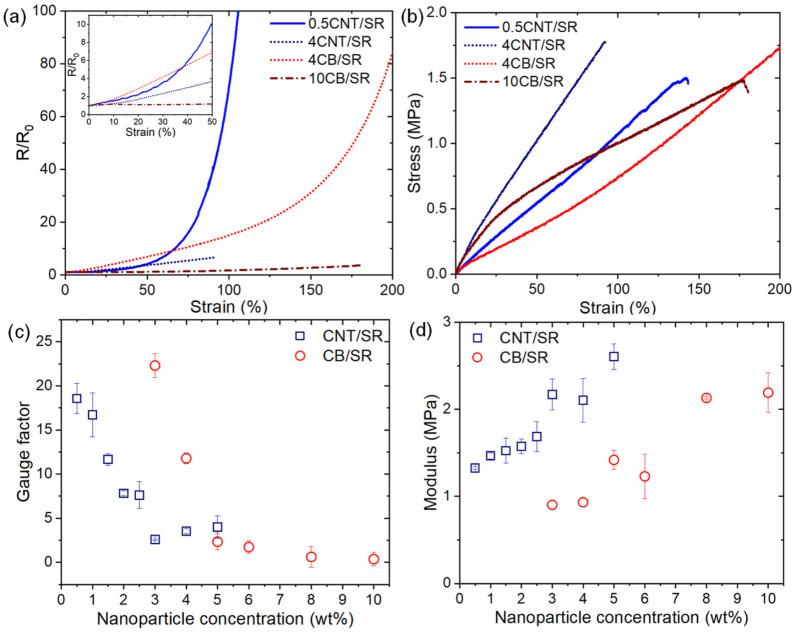
(**a**) Monotonic strain sensing behaviors and (**b**) stress–strain curves of the four typical composites (0.5CNT/SR, 4CNT/SR, 4CB/SR, 10CB/SR). (**c**) Gauge factor (**d**) modulus as a function of the NPs loading.

Figure 3a,b show the normalized resistance change and stress–strain curves of the four representative samples when stretched. These four samples exhibit similar mechanical properties, but the strain sensitivity is different. The 0.5CNT/SR and 4CB/SR composites exhibited a steady increase in resistance under monodirectional loading, indicating a distinct electro-mechanical response. While the 4CNT/SR and10CB/SR, far from the percolation threshold, are less strain-sensitive. In addition, as the inset in Figure 3a shows, when the strain is less than 50%, *R*/*R*_0_ of the 0.5CNT/SR composite exhibits an exponentially increasing tendency. In contrast, the other three composites show a nearly linear increasing tendency. Similar phenomena have been reported in the literature and have been attributed to the dominant conduction mechanism. When the NPs content is low, the tunneling resistance increases exponentially with strain, and the dominant conduction mechanism transforms to contacting conduction with the increase of NPs concentration and shows linear behavior [34,38]. As shown in Figure S2, we compared the influence of stretch speed on the mechanical and resistive response of CPCs. Generally, the rate-dependent viscoelasticity is not profound, as indicated by the similar response of both mechanical and resistive responses at different rates.

The sensitivity magnitude is commonly referred to as the gauge factor (GF), *s*, which is defined by:

(3) $$s=\frac{\Delta R/R_{0}}{\varepsilon },$$

where ΔR is the resistance change, *R*_0_ is the initial resistance, and ε is the tension strain. Here, according to Equation (3), GF is determined based on a strain of 50%. As shown in Figure 3c, as GF decreases with the increasing of NPs loading, it is evident that the CPCs with NPs contents at the vicinity of the percolation threshold showed higher GF than the CPCs with higher NPs contents. This is consistent with the well-known strain sensing property that high resistive sensitivity is usually achieved when the conductive filler content is slightly above the percolation threshold because of the vulnerable conductive networks to external stimuli [19,39]. In addition, the GF of the 0.5CNT/SR and 3CB/SR composites is about 18.6 and 22.3, respectively.

Furthermore, when the NPs loading is well above the percolation threshold, i.e., CNT loading ≥ 3 wt% and CB loading ≥ 5 wt%, the GF almost does not change with NPs content. The significant reduction in GF is accounted for in the efficient compensation of tunneling resistance changes and robust rearrangement of the dense conductive networks [26]. Figure 3d shows the evolution of Young’s moduli as a function of the NPs loading. Here, Young’s modulus is determined from the initial slope of the nominal stress–strain curve (at 10% strain), and the modulus of both CNT/SR and CB/SR composites is less than 3 MPa. It is clear that higher filler content results in a higher Young’s modulus, which is due to the reinforcement of CPCs by NPs. In addition, the modulus of CB/SR composite is smaller than that of the CNT/SR composite with the same filler content, making it more suitable for soft sensors. It should be noted that, in addition to electrical and mechanical properties, environmental factors such as humidity should also be characterized to ensure that the CPCs have good environmental stability [40].

### 3.3. Effect of Filler Content on Resistive Hysteresis of CNT/SR and CB/SR

**Figure 4:**
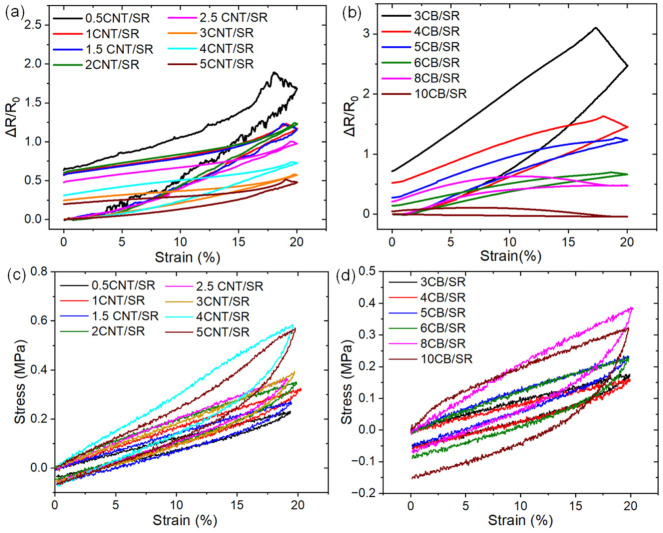
The relative resistance *R*/*R*_0_ of the CNT/SR (**a**) and CB/SR (**b**) composites during the first loading/unloading cycle at 20% strain. The stress of the CNT/SR (**c**) and CB/SR (**d**) composites during the first loading/unloading cycle at 20% strain.

The loaded/unloaded test was conducted to evaluate the effect of NPs dimensionality and content on the resistive hysteresis, where the maximum strain ($\varepsilon_{max}$) was 20%. Due to the high reorganization of the polymer chains and NPs network during the first cycle, the mechanical and resistive hysteresis during the first loading/unloading cycle is higher than in subsequent cycles [41,42]. Therefore, we focused on the relative resistance *R*/*R*_0_ and stress change of the CPCs in the first loaded/unloaded cycle. As shown in Figure 4a,b, *R*/*R*_0_ increases during stretching, continues to increase, and then decreases during release, and cannot recover to its initial value after unloading to zero strain. The mechanism for the resistance increasing upon release will be discussed later on. Evidently, the lower the NPs content, the more significant the increase in *R*/*R*_0_ upon loading and the higher the residual *R*/*R*_0_ after unloading. Moreover, for the 0.5CNT/SR composites, fluctuation in resistance can be seen on the loading/unloading curve, indicating the occurring of a fast and irreversible process such as conductive network rearrangement by CNT-to-CNT sliding within the composite [13]. Figure 4c,d summarize the stress–strain curves of CPCs with different NPs content during loading/unloading cycle; it can be seen that the maximum stress at $\varepsilon_{max}$ increases significantly with NPs loading, contrary to the maximum resistance decreasing with the increase of NPs.

**Figure 5:**
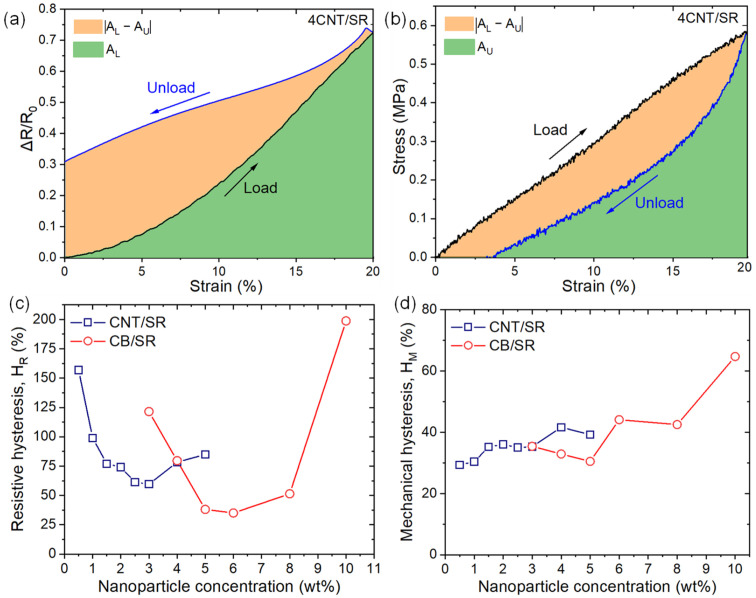
The calculation diagram of the (**a**) resistive hysteresis (*H_R_*) and (**b**) mechanical hysteresis (*H_M_*) ; (**c**) *H_R_* and (**d**) *H_M_* as a function of NPs contents.

To quantitatively describe resistive hysteresis (*H_R_*), we calculated *H_R_* in a similar fashion to mechanical hysteresis (*H_M_*) as follows [10,25,41]:

(4) $$H_{i}=\frac{\left|A_{L}-A_{U}\right|}{A_{L}},$$

where $A_{L}$ and$\;A_{U}$ are the areas under the loading and unloading curves, respectively. $\left|A_{L}-A_{U}\right|$ is the area between the loading and the unloading curves. As Figure 5a,b show, *i* = *R* for the resistive hysteresis (Δ*R*/*R*_0_ vs. *ε*) and *i* = *M* for the mechanical (*σ* vs. *ε*) one. It should be noted that, on the resistance–strain curve, the unloading curve is above the loading curve, while on the stress–strain curve, the unloading curve is below the loading curve. The resistive hysteresis and mechanical hysteresis of CPCs are summarized in Figure 5c,d as a function of CNT and CB content. Overall, the resistive hysteresis decreases with NPs content at low concentrations above percolation thresholds while further increasing with NPs content at higher NPs loading. 3CNT/SR and 6CB/SR have the lowest resistive hysteresis of 59.6% and 35.0% in CNT and CB-filled CPCs, respectively. In comparison, the mechanical hysteresis tends to increase with increasing NPs loading, which is consistent with the well-known conclusion that an increased filler fraction in rubbers leads to higher mechanical hysteresis [25].

**Figure 6:**
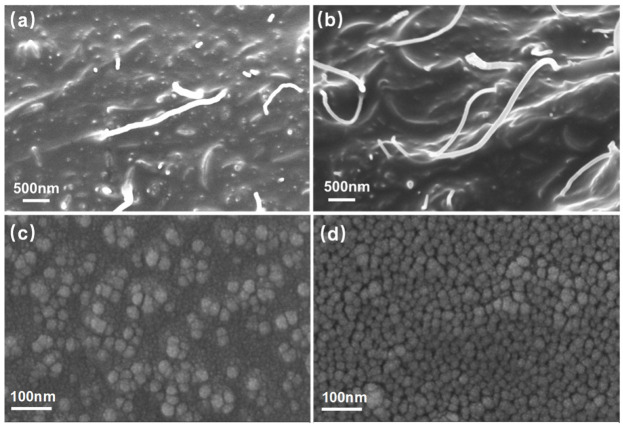
SEM images of fracture surface of (**a**) 0.5CNT/SR, (**b**) 4CNT/SR, (**c**) 4CB/SR, and (**d**) 10CB/SR composite samples.

The resistive hysteresis is ascribed to the strain-induced reorganization of the electrically conductive network and viscoelastic ingredients of materials [20,41]. In the NPs-filled elastomers, the average strain in the elastomeric domains is necessarily amplified over that of the macroscopic strain because the stiff particles accommodate little of the macroscopic strain, and the amplification factor X is dependent on particle volume fraction and distribution [43]. Figure 6 shows the zoom-in of the fracture surface, and it can be seen that the CNT density of the 0.5CNT/SR composite is lower than that of the 4CNT/SR composite (Figure 6a,b), and the CB density of the 4CB/SR composite is lower than that of the 10CB/SR composite (Figure 6c,d). Therefore, the nanocomposite consisting of high NPs tends to form a dense conductive network. It is believed that the resistive hysteresis is caused by the permanent destruction of a portion of the unstable conductive networks and the hysteresis effect of rubber under cyclic loading [7]. Therefore, it can be concluded that, when NPs concentration approaches the percolation thresholds, the resistive hysteresis is mainly caused by the permanent destruction of the unstable conductive networks. In contrast, when NPs concentration is high, resistive hysteresis may be mainly caused by the Mullins effect due to the internal friction between the polymer network and NPs [2].

### 3.4. Effect of Filler Content on Resistance Relaxation of CNT/SR and CB/SR

**Figure 7:**
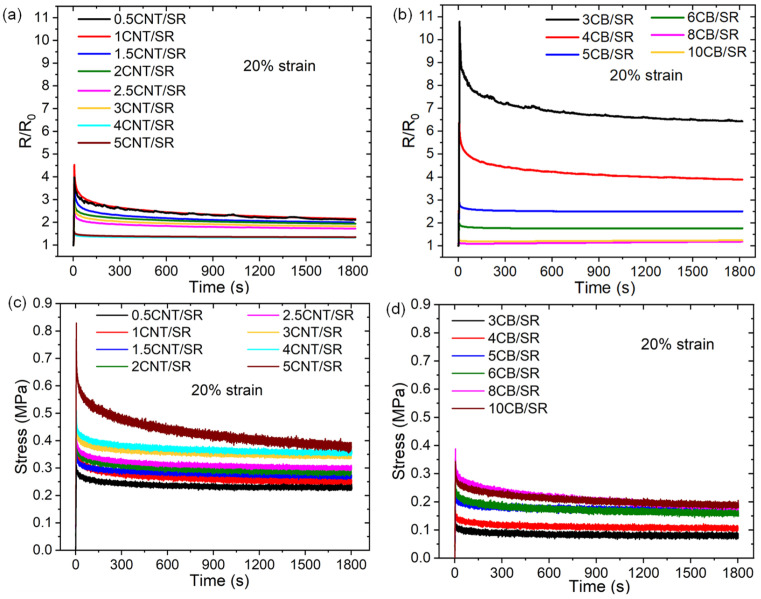
The relative resistance *R*/*R*_0_ of the CNT/SR (**a**) and CB/SR (**b**) composites as a function of time *t* during relaxation experiments at 20% strain. The stress of the CNT/SR (**c**) and CB/SR (**d**) composites as a function of time *t* during relaxation experiments at 20% strain.

Soft strain sensors are inevitably used for continuous monitoring in long-term applications. Resistance attenuation also occurs during long-term stress relaxation, which is thought to be caused by the deformation of polymer chains [10]. The effects of filler type and filler concentration on the resistance relaxation of CPCs were investigated, and the corresponding stress relaxation was compared. Relaxation experiments were conducted at three strain levels of 10%, 20%, and 30%, as shown in Figure 7, taking the relaxation experiment at the 20% strain level as an example. Overall, the stress and resistance relaxation show the same tendency, i.e., the stress/resistance initially shows an abrupt jump and reaches maximum stress/resistance, then decreases with time *t* to reach a new equilibrium value. The increase in particle-to-particle spacing increases the resistance during stretching, while the alignment of anisotropic particles along the direction of strain decreases the resistance during relaxation [44]. NPs concentration has an opposite effect on resistance/stress relaxation, i.e., the maximum *R*/*R*_0_ and equilibrium *R*/*R*_0_ decrease with NPs concentration, while the maximum stress and equilibrium stress increase with NPs concentration.

We evaluated stress relaxation using the relaxation ratio, which is defined as [45]:

(5) $$r_{\sigma }=\frac{\sigma_{I}-\sigma_{e}}{\sigma_{I}},$$

where $\sigma_{I}$ is the initial stress that begins to attenuate, $\sigma_{e}$ is the equilibrium stress. The experimental results show that resistance relaxation and stress relaxation exhibit a similar variation trend. Therefore, the resistance relaxation ratio was also defined as [45]:

(6) $$r_{R}=\frac{R_{I}-R_{e}}{R_{I}},$$

where *R_I_* is the initial resistance that begins to attenuate, which is also the maximum *R*/*R*_0_ on the resistance relaxation curve, *R_e_* is the equilibrium resistance. The resistance/stress relaxation data of CNT/SR and CB/SR at the three strain levels of 10%, 20%, and 30% are listed in Table 1 and Table 2, respectively. One can see that the NPs loading also has an opposite effect on the resistance relaxation ratio and stress relaxation ratio, i.e., the resistance relaxation ratio decreases with the increase of NPs loading. In contrast, the stress relaxation ratio increases with NPs loading. In addition, the resistance and stress relaxation ratios enhanced with strain levels. Therefore, the resistance relaxation reflects the conductive network degradation, and a dense conductive network (with high NPs content) results in a smaller resistance relaxation ratio despite larger stress relaxation.

### 3.5. Effect of Filler Content on Dynamic Resistance Response of CNT/SR and CB/SR

**Figure 8:**
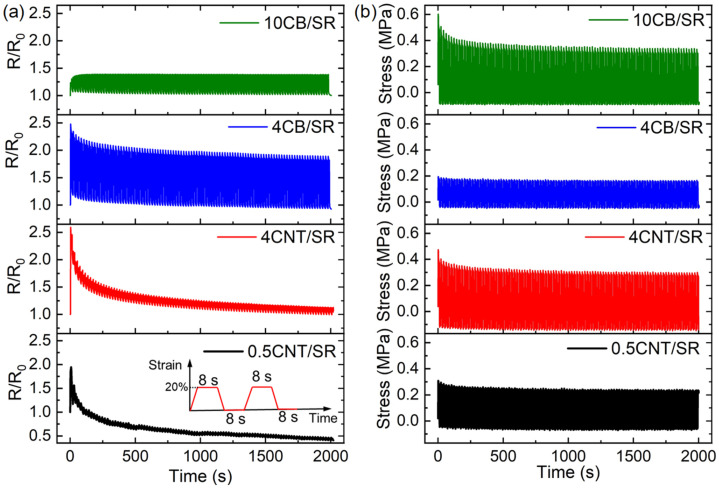
Relative resistance *R*/*R*_0_ (**a**) and stress (**b**) change of CPCs during 100 cyclic loaded/unloaded test as functions of NPs and time. The inset shows the loading history for the dynamic cyclic loading test with a maximum strain of 20%.

Characterizing cyclic electro-mechanical behavior is essential to evaluating the reproducibility and reliability of soft strain sensors. As the inset in Figure 8a shows, the CPCs were loaded/unloaded for 100 cycles at a maximum strain of 20%, and stress relaxation was tested during loading/unloading cycles where samples were loading/unloading at a strain rate of 0.1 s^−1^ with intermittent 8 s strain holding periods during both loading and unloading.

#### 3.5.1. Resistance and Stress Response

Figure 8a shows the relative resistance responses *R*/*R*_0_ vs. time of four representative CPCs of 0.5CNT/SR, 4CNT/SR, 4CB/SR, and 10CB/SR during the loading/unloading cycle. It can be seen that the resistance oscillates in each cycle. Obviously, for the CNT-filled composites of 0.5CNT/SR and 4CNT/SR, the maximum and minimum *R*/*R*_0_ values after loading and unloading gradually decrease with increasing cycles. For the 4CB/SR composites, the maximum and minimum *R*/*R*_0_ values decrease at the beginning and reach a steady state in subsequent cycles. Nevertheless, for the 10CB/SR composites, the maximum *R*/*R*_0_ increases in the first few cycles and remains unchanged in subsequent cycles. In contrast, the minimum *R*/*R*_0_ was maintained as almost constant throughout the process. It is assumed that strain conditioning occurred in the first few cycles, where most of the weak or unstable parts in conductive networks have vanished, so subsequent cycles produce more reproducible resistance changes [26]. In agreement with previous reports, the strain-dependent response for CB-filled composite was relatively steady in the durability test, while the response of CNT-filled composite slightly drifted and fluctuated [29].

When the test is suspended during loading and unloading, time dependence in the stress response will be observed [43]. It is considered that the observed drift/decay of most piezoresistive composite materials under dynamic loading is a direct consequence of the relaxation from polymer chains and conductive networks [22]. Here, we compare the stress–time curves of the four representative samples during cyclic tests. Figure 8b shows that the minimum stress remains unchanged during loading/unloading cycles. For the 4CNT/SR and 10CB/SR composites, the maximum stress decreases during the first few cycles and maintains its value in the following cycles. However, for the 0.5CNT/SR and 4CB/SR samples, the maximum and minimum stresses remain almost the same during cycling. The monotonic and stable stress change during cyclic loading suggested that the influence of the conductive network on the resistance response is greater than that of the viscoelasticity of the polymer matrix. Therefore, we will focus on the resistance response of the CPCs and analyze it from the perspective of conductive networks.

#### 3.5.2. Decline of Sensitivity

**Figure 9:**
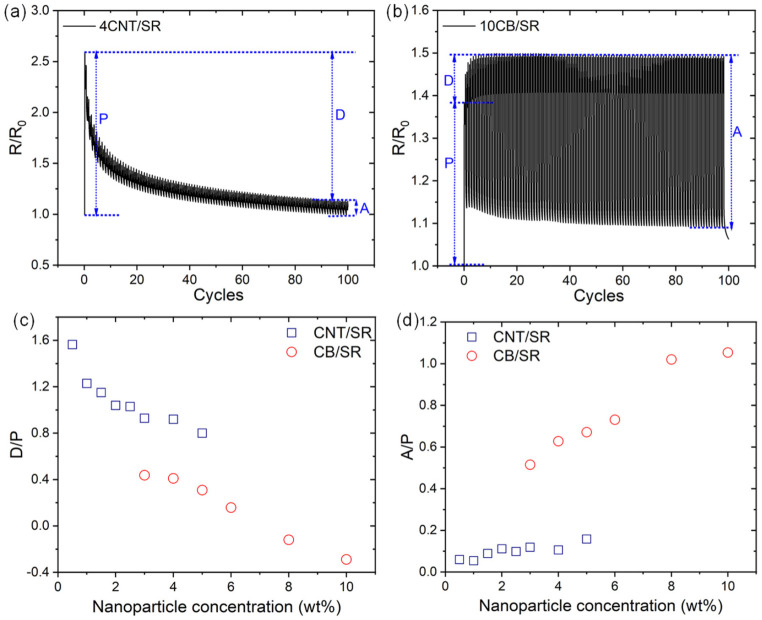
Schematic diagrams of obtaining the peak of the first cycle P, the decrease/increase of peak D, the change amplitude A for CPCs during 100 cyclic loaded/unloaded test, taking 4CNT/SR (**a**) 10CB/SR (**b**) samples as an example. D/P (**c**) and A/P (**d**) as a function of NPs loading.

Maintaining a high sensitivity of CPCs during cyclic stretching and relaxation is as important as obtaining high initial sensitivity. As shown in Figure 9a,b, we introduced the relative resistance *R*/*R*_0_ peak of the first cycle (P), the decrease of peak value during cyclic strains (D), and the change amplitude (A) of *R*/*R*_0_ for CPCs to evaluate the decline of sensitivity [17,46]. The D/P represents the attenuation ratio, A/P describes the maintenance of sensitivity, and the increase in D/P and the decrease in A/P both represent the decline of sensitivity during cycling. These two parameters of CPCs with different filler content and type are calculated and listed in Figure 9c,d. It should be noted that the peak values D of the 8CB/SR and 10CB/SR composites increase during the first few cycles; thus, it is negative (as shown in Figure 9b). It can be seen that the lower the NPs loading, the higher the attenuation ratio D/P, and the smaller the sensitivity A/P, which means a greater decline of sensitivity during cyclic loading/unloading. The conductive network rearrangement capability for CNT and CB composites is different. The high aspect ratio CNT is more effective in forming conductive pathways while compromising mobility during dynamic deformation. By contrast, nanoscale CB particles have higher mobility and, thus, better network reformation capability under cyclic deformation. Due to the above reasons, the CB/SR composites show a smaller sensitivity decline during cyclic loading/unloading than CNT/SR composites, corresponding to higher reliability.

#### 3.5.3. Secondary Peaks

**Figure 10:**
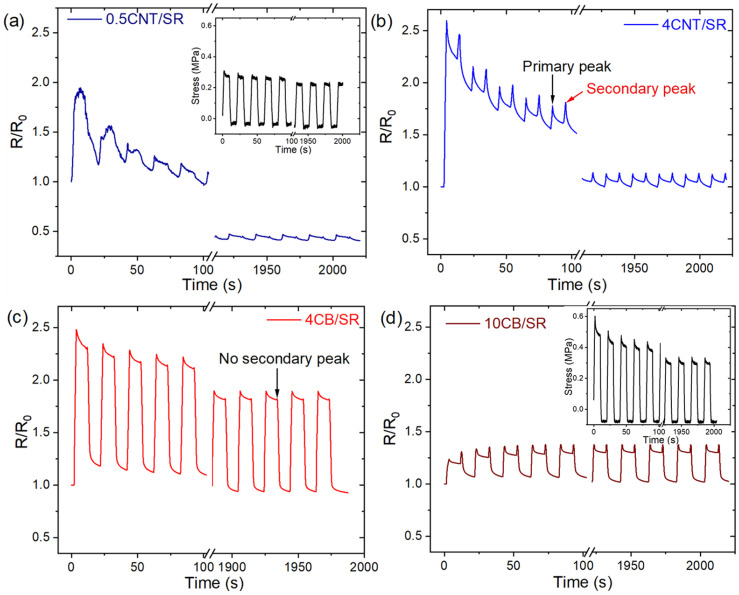
The effect of NPs contents and dimensionality on secondary peaks, focusing on the first 5 cycles and the last 5 cycles: (**a**) 0.5CNT/SR, (**b**) 4CNT/SR, (**c**) 4CB/SR, (**d**)10CB/SR. The inset shows the corresponding stress-time curves.

The cyclic resistance response of CPCs generally exhibits the shoulder phenomenon or secondary peaks, i.e., in addition to the resistance peak at maximum strain, additional peaks either occur while releasing or when the imposed strain is fully released [16]. For the sake of clarity, we selected the beginning/last five cycles of 0.5CNT/SR, 4CNT/SR, 4CB/SR, and 10CB/SR during loading/unloading, as summarized in Figure 10. During both loading and unloading, the resistance was observed to decrease during the strain hold period. This was different from the mechanical response, which reduces during the strain-holding period of loading, and increases during the strain-holding period of unloading (as the insert figures shown in Figure 10a,d). In addition, CPCs show lower secondary peaks when NPs content is lower, and secondary peaks are higher than the primary peaks when NPs content is higher. In addition, surprisingly, the 4CB/SR sample has almost no secondary peaks. It has been reported that these shoulder phenomena are caused by the re-distributions, reconstruction, and simultaneous destruction of the electrically conductive networks [8].

**Figure 11:**
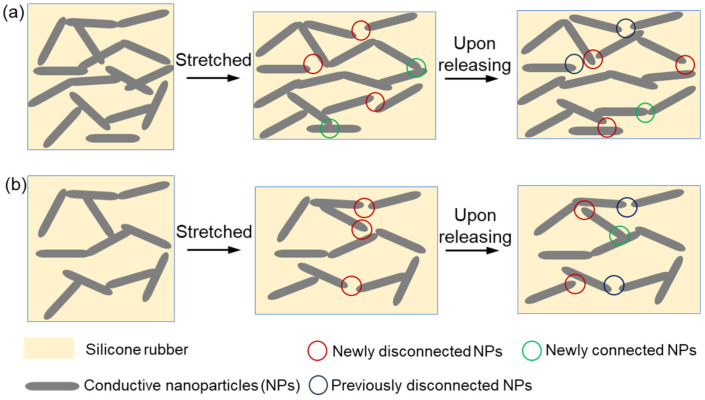
Proposed mechanism for structural transition of stretched conductive network upon stress release for CPCs: (**a**) dense conductive network (high NPs concentration), (**b**) loose conductive network (low NPs concentration).

The high secondary peak upon stretching recovery was prominent for composites with high NPs loading. We attribute this increase of resistance of the second peak to the structural recovery mismatch of the NPs and matrix, i.e., faster recovery of the deformed NPs in the conductive network than the polymer matrix network, as illustrated by the schematics in Figure 11. At higher particle loading, the stretching of the dense conductive networks formed many new conductive pathways by the alignment of highly elastic NPs, as revealed by a slower increasing rate or even a drop in resistance at strains over 10% (Figure 4b). Upon release, the instant removal of the lateral compression drives the fast recovery of the deformed NPs while the polymer network maintains a stretched state due to delayed contraction. The elastic recovery of NPs will disconnect some newly formed conductive pathways, leading to an instant drop in conductivity and an abrupt rise in resistance as the second peak (Figure 11a). Note this phenomenon is less prominent for composites with lower particle loading due to fewer newly formed conductive pathways by stretching and, thus, a weak change in the conductivity (Figure 11b).

Overall, compared with CNT/SR composites, the resistance response of CB/SR composites with low NPs content exhibits better repeatability and stability in resistance response during dynamic cyclic loading, making them more suitable for the fabrication of soft strain sensors.

## 4. Conclusions

In this paper, we have demonstrated that the NPs’ dimensionality and content can significantly affect the resistive viscoelasticity properties of CPCs. A percolation threshold of ca. 0.2 wt% of CNT/SR, which is much lower than that of 2.0 wt% of CB/SR, is obtained. The gauge factors of the 3CB/SR composite and 0.5CNT/SR composite were 20.3 and 18.6, respectively, and decreased with the increasing of NPs loading. Based on the SEM images as well as the electrical properties, the good dispersion of CB and CNT fillers in SR matrix is confirmed, which ensures the reliability of CPCs in the study of electro-mechanical properties.

The electro-mechanical testing results showed a prominent decline in sensitivity (as indicated by GF), resistive hysteresis ratio, and resistance relaxation ratio above the percolation thresholds for the CPCs at low NPs contents. However, the declined maximum stress during dynamic cyclic loading, the stress hysteresis, and the stress relaxation ratio all increased with the increase in NPs concentration. When approaching the percolation thresholds, the resistive viscoelasticity mainly originated from the decay of the conductive network; while at higher NPs concentration, the capability of conductive network rearrangement and their interactions with the polymer network matrix significantly govern the mechanical and resistance response. In addition, higher NPs loading leads to higher secondary peaks for both CB/SR and CNT/SR composites, and the secondary peaks for CB/SR composite are negligible when the CB concentration is low. For CPCs-based soft strain sensors, the resistive viscoelasticity properties should be characterized in addition to the sensitivity when selecting the recipe for NPs filler. Compared with one-dimensional CNT-filled CPCs, the zero-dimensional CB-filled CPCs exhibit higher sensitivity, lower resistive hysteresis, lower resistance relaxation ratio, and better cyclic performance, thus are more suitable for sensor usage. Like the mechanical viscoelasticity, the strain rate plays a critical role in the resistive viscoelasticity. Future work is needed to develop a more sophisticated model with which to better understand the rate dependence of CPCs resistance. The present study provides a guideline for fabricating CPCs-based soft strain sensors with low resistive viscoelasticity by simply adjusting the dimensionality and content of NPs.

## Figures and Tables

**Table 1:** The resistive/stress relaxation ratio of CNT/SR as a function of CNT content and strain level.

CNT (wt%)	Resistive Relaxation Ratio (R_I_ − R_e_)/R_I_ (%)			Stress Relaxation Ratio (σ_I_ − σ_e_)/σ_I_ (%)		
	10%	20%	30%	10%	20%	30%
0.5	44.7	48.3	73.2	15.5	25.9	23.8
1	39.7	52.5	47.6	15.8	29.9	24.4
1.5	40.3	43.9	47.4	18.8	29.1	29.6
2	32.1	37.3	38.4	19.3	30.9	36.7
2.5	28.0	33.6	39.9	23.1	31.6	30.0
3	19.1	34.8	32.8	28.0	29.8	35.9
4	13.9	14.8	16.3	30.1	32.3	44.5
5	9.23	15.2	14.2	27.1	52.7	45.1

**Table 2:** The resistive/stress relaxation ratio of CB/SR as a function of CB content and strain level.

CB (wt%)	Resistive Relaxation Ratio (R_I_ − R_e_)/R_I_ (%)			Stress Relaxation Ratio (σ_I_ − σ_e_)/σ_I_ (%)		
	10%	20%	30%	10%	20%	30%
3	20.7	40.8	45.4	16.7	17.8	22.1
4	13.4	38.8	47.9	18.7	19.6	23.5
5	13.7	13.2	23.2	19.6	20.9	26.1
6	10.8	10.9	16.6	22.0	28.0	28.4
8	5.04	3.62	4.46	31.9	34.4	41.4
10	6.01	3.15	3.55	38.7	33.6	55.8

## Data Availability

The data presented in this study are available on request from the corresponding author.

## Supplementary Materials

The following supporting information can be downloaded at: https://www.mdpi.com/article/10.3390/polym15163379/s1, Figure S1: The true stress-strain curves and monotonic strain sensing behaviors of CNT/SR and CB/SR composites; Figure S2 The effect of stretch speed on (a) true stress-strain behavior and (b) resistance response.
