# Supplementary material for: The Effect of Filler Dimensionality and Content on Resistive Viscoelasticity of Conductive Polymer Composites for Soft Strain Sensors
Source: Polymers (Basel). 2023 Aug 11;15(16):3379. doi: 10.3390/polym15163379 (PMC10458475; doi:10.3390/polym15163379)
Supplement: Supplementary file 1 [file polymers-15-03379-s001.zip › polymers-2503785-supplementary.pdf]

Content

Figure S1 The true stress-strain curves and monotonic strain sensing behaviors of CNT/SR and CB/SR composites.

Figure S2 The effect of stretch speed on (a) true stress-strain behavior and (b) resistance response.

The true stress-strain curves and relative resistance-strain curves for the CPCs were summarized in Figure S1. The nominal strain is calculated as [25]:

$$\varepsilon_{nom} = \Delta L/L_0, \tag{S1}$$

where L_0 is the gauge length, ΔL is the crosshead displacement corresponding to the undeformed state. The true strain λ is calculated as:

$$\lambda = \ln\left(1 + \frac{\Delta L}{L_0}\right), \tag{S2}$$

The nominal stress, σ_{nom} is defined as:

$$\sigma_{nom} = F/S_0 \tag{S3}$$

where F is the measured force and S_0 is the initial cross-sectional area of the specimen. Considering iso-volume deformation conditions, the true stress σ_t can be expressed as:

$$\sigma_t = \sigma_{nom}(1 + \varepsilon_{nom}) \tag{S4}$$

As shown in Figure S2, we compared the influence of stretch speed on the mechanical and resistive response of CPCs.

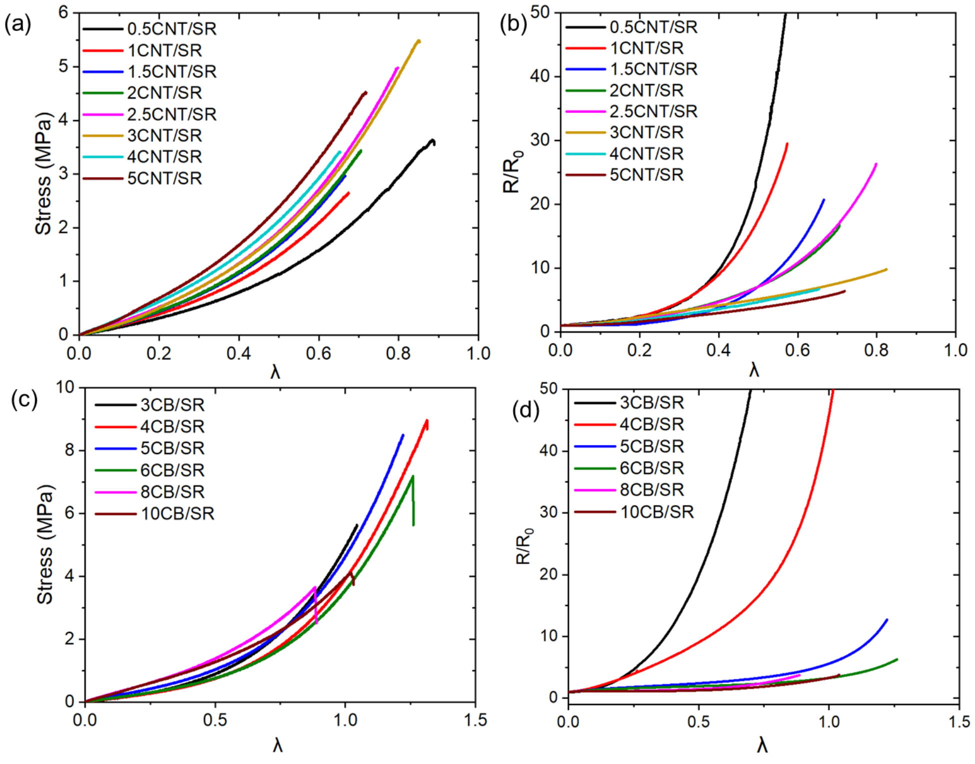

Figure S1. (**a**) The true stress-strain curves of CNT/SR; (**b**) Monotonic strain sensing behaviors of CNT/SR; (**c**) The true stress-strain curves of CB/SR; (**d**) Monotonic strain sensing behaviors of CB /SR.

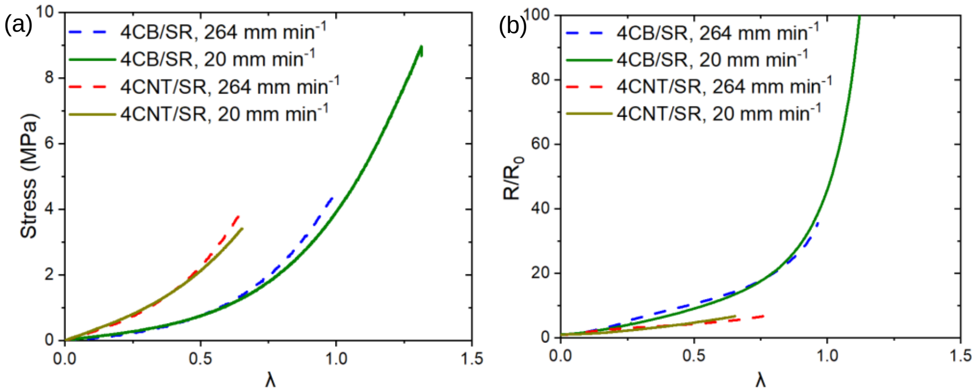

Figure S2. The effect of stretch speed on (**a**) true stress-strain behavior and (**b**) resistance response.
